# Acute Inflammatory Demyelinating Polyneuroradiculopathy with Ipilimumab in Metastatic Melanoma: A Case Report and Review of Literature

**DOI:** 10.7759/cureus.1310

**Published:** 2017-06-05

**Authors:** Chintan Rupareliya, Syeda Naqvi, Vishal B Jani

**Affiliations:** 1 Department of Neurology, University of Missouri, Columbia, Missouri; 2 Jinnah Postgraduate Medical Centre, Jinnah Sindh Medical University (SMC); 3 Depaetment of Neurology, Creighton University

**Keywords:** ipilimumab, metastatic melanoma, immune checkpoint inhibitors, aidp

## Abstract

Ipilimumab (Bristol-Myers Squibb Co., New York, NY) is a novel anticancer medication used for the treatment of metastatic melanoma. The exact mechanism of its action remains unclear; however, data from previous clinical trials postulates the immunomodulatory activity of ipilimumab to enhance therapeutic effectiveness. Ipilimumab was approved by the Food and Drug Administration (FDA) in March 2011 for use in stage III and IV of unresectable metastatic melanoma.

We report a single case of acute inflammatory demyelinating polyneuroradiculopthy (AIDP) in the patient treated with ipilimumab for recurrent metastatic melanoma. The patient presented with multiple falls that started after the third infusion of ipilimumab. Other symptoms were hoarseness of voice, motor deficits in his right arms, and tingling in both hands. The deficits progressed into near complete loss of movement and sensation in all four extremities over the course of two weeks. However, his bladder and bowel functions were intact. There was no history of fever, recent travel, exposure to sick contacts, insect bites, or gastrointestinal symptoms.

Along with strong immune-mediated pharmacological response towards cancer cells, ipilimumab also induces immune-related adverse events (irAEs) within normal tissues by the mechanism of molecular mimicry.

## Introduction

Ipilimumab is a human monoclonal antibody directed against cytotoxic T-lymphocyte-associated antigen four (CTLA-4). By upregulating an immune reactivity to cancer, ipilimumab works by suppressing T-lymphocyte-mediated immune inhibition resulting in the enhancement of immune-mediated response against tumor cells. Another drug with a similar mechanism is nivolumab, a monoclonal antibody directed against programmed cell death one (PD-1) receptors [1]. Along with an enormous pharmacological response elicited towards cancer cells, ipilimumab causes immune-related adverse events (irAEs) within normal tissues. Most common irAEs include gastrointestinal, endocrine, skin, and liver toxicities [2]. The nervous system is rarely involved. irAEs include temporal arteritis, Guillain-Barre syndrome (GBS) or AIDP, sensorimotor neuropathy, severe meningo-radiculoneuritis, aseptic meningitis, and posterior reversible encephalopathy syndrome [2]. This is a rare case because very few cases of acute inflammatory demyelinating polyneuroradiculopthy (AIDP) complicating ipilimumab treatment have been reported in the past [1, 3].

## Case presentation

A 77-year-old right-handed man undergoing ipilimumab treatment for recurrent metastatic melanoma presented with multiple falls that started three days after his third ipilimumab infusion. The symptoms began with hoarseness of voice, right arm weakness, and tingling in the bilateral hands which progressed over the next two weeks to near complete loss of movement and sensation in all four extremities. The patient had no bowel or bladder incontinence. He also denied any fever, sick contacts, recent travel, insect bites, or gastrointestinal symptoms.

On exam, his mental status and all cranial nerves were intact except for significant hoarseness and hypophonia. The tone was flaccid in his distal extremities and decreased proximally but no atrophy was present. Proximal strength was reduced overall in all extremities except during left shoulder abduction, left elbow flexion, and left elbow extension. Sensation was decreased in all extremities (distal > proximal). Reflexes were also absent in all extremities. Toes were mute to plantar stimulation bilaterally.

**Figure 1:**
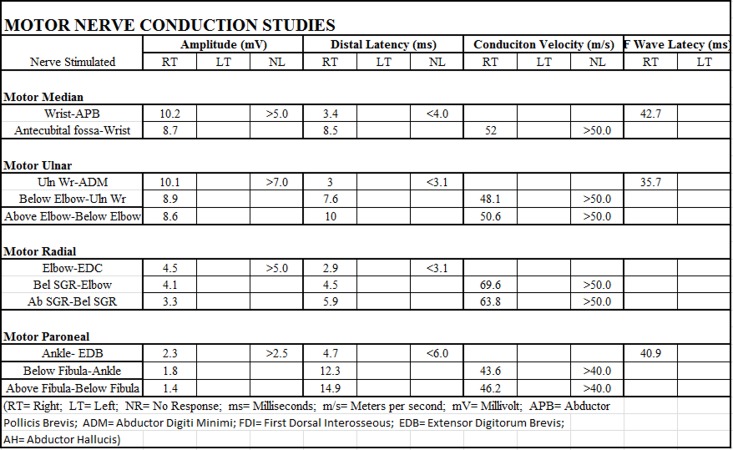
Electromyogram on the seventh day after symptom onset RT = Right, LT = Left, NR = No Response, ms = milliseconds, m/s = meters per second, mV = millivolt, APB = Abductor Pollicis Brevis, ADM = Abductor Digiti Minimi, Uln = Ulnar, Wr = Wrist, EDC = Extensor Digitorum Communis, EDB = Extensor Digitorum Brevis, Ab = Above, Bel = Below

**Figure 2:**
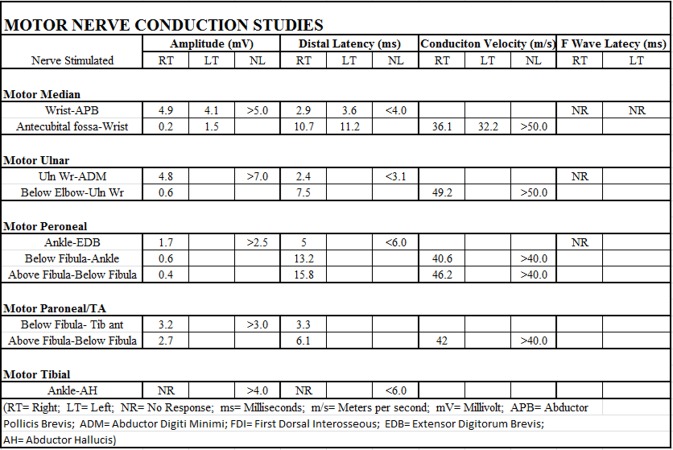
Electromyogram on the twenty-first day after symptom onset RT = Right, LT = Left, NR = No Response, ms = Milliseconds, m/s = Meters per second, mV = Millivolt, APB = Abductor Pollicis Brevis, ADM = Abductor Digiti Minimi, FDI = First Dorsal Interosseous, EDB = Extensor Digitorum Brevis, AH = Abductor Hallucis, Uln = Ulnar, Wr = Wrist, Tib = Tibula

A magnetic resonance imaging (MRI) scan of the entire spine with and without contrast was negative for metastatic disease, cord compression, cord edema, or other etiology. The MRI brain scan with and without contrast was also negative for malignancy or any other acute pathology. The first electromyogram (EMG) of the right upper and lower extremity is shown in Figure 1 which was done seven days after the onset of the symptoms (ten days after his last ipilimumab dose). The EMG demonstrated no evidence of definite myopathy. In the right upper extremity, there was evidence of chronic neurogenic changes as well as decreased recruitment of all muscles tested. In the right lower extremity, there was a concern for polyneuropathy or polyradiculopathy. Repeat EMG shown in Figure 2 was done 21 days after the onset of the symptoms and 24 days after his last ipilimumab/nivolumab dose. It demonstrated electrophysiologic evidence consistent with AIDP with secondary axonal features. 

The cerebrospinal fluid (CSF) analysis showed elevated immunoglobulin G (IgG). The serum paraneoplastic panel was negative except for high titers of striatal antibody. Other pertinent serum findings were elevated glutamic acid decarboxylase (GAD65), negative IgG and immunoglobulin M (IgM) for Lyme disease, elevated erythrocyte sedimentation rate (ESR), normal C-reactive protein (CRP), normal creatine kinase (CK) and elevated myoglobin. Because of hoarseness in his voice, there was a concern for bulbar dysfunction which was evaluated by speech pathologists and ear, nose, and throat (ENT) service. Swallow evaluation and modified barium swallow were also performed. They demonstrated moderate to severe oropharyngeal dysphagia complicated by trace penetration and silent aspiration with all liquid consistencies. The ENT service also conducted a bedside flexible fiberoptic nasolaryngoscopy which demonstrated reduced motor and sensory function of the larynx, poor glottic closure, and right vocal cord weakness.

## Discussion

Ipilimumab is a human monoclonal antibody used for the treatment of recurrent metastatic melanoma. This novel drug has been shown to improve overall median survival in patients with pretreated melanoma [1]. Ipilimumab is directed against CTLA-4 causing immunomodulation. Previous studies have shown increased levels of T-lymphocytes directed towards axonal gangliosides in the pathogenesis of GBS which may also explain the incidence of GBS from ipilimumab-induced continued T lymphocyte proliferation. In order to understand the immunomodulatory action of ipilimumab, it is important to first know the normal T-cell response. Tumor cells carry antigens on their surface which are binding sites for T-cell receptors (TCR) and CD 28. TCR and CD 28 are present on T-cell surface. Binding of CD 28 with B7 antigens on antigen presenting cells (APC) initiatesT-cell proliferation. In this phase, CTLA-4 (a naturally occurring inhibitor) receptors are upregulated and migrated towards the cell surface. Upon reaching the cell surface, CTLA-4 binds to B7 with a higher affinity than CD 28, ceasing the process of T-cell proliferation. By use of ipilimumab, no free binding sites are available for B7 and hence inhibition of T-cell proliferation does not happen. Continued T-cell proliferation results in regression of metastatic lesions. A similar mechanism is also responsible for immune-related adverse events from ipilimumab [2]. Our case is rare because very few cases of GBS complicating ipilimumab therapy have been reported previously in the medical literature [1, 3].

Other drugs with a similar mechanism are nivolumab and pembrolizumab. They are monoclonal antibodies that bind to PD-1 receptors and prevent T-cell inactivation. One trial of ipilimumab and nivolumab combination showed that they are also very effective but the likelihood of adverse events is also cumulative [4]. An incidence of irAEs from nivolumab alone is much less than with ipilimumab; nivolumab causes increased T-cell production by preventing PD-1 receptors to bind with its ligands - programmed death-ligand 1 (PD-L1) and programmed death-ligand 2 (PD-L2) [4]. Apart from rendering an antitumor effect, this mechanism also potentiates aberrant humoral immune response towards gangliosides. Molecular mimicry is very well understood by the pathologic mechanism which could explain the damage to normal tissues (by accelerated T-lymphocyte response) with ipilimumab therapy. A study that examined the time to onset and the resolution of irAEs from ipilimumab therapy showed that the most commonly affected systems are the skin and the GI tract. One study also showed that the majority of irAEs occurs during an induction phase (first 12 weeks after beginning therapy) with the median time of onset being six weeks [4].

Although infrequent, the nervous system is profoundly affected by the on-going T-cell proliferation. Few other cases of nervous system involvement are myasthenia gravis, transverse myelitis, hypophysitis, chronic inflammatory demyelinating polyneuroradiculopathy (CIDP), and bilateral facial palsy [5-7].

Compared to the previous cases, ours was more serious evident by complete immobilization, worsening bulbar involvement requiring percutaneous endoscopic gastrostomy (PEG), and longer intensive care unit (ICU) stay. Prompt withdrawal of ipilimumab (after the diagnosis of GBS) was done and the patient was initially administered five cycles of intravenous immunoglobulin (IVIG), with only mild improvement in weakness and paresthesia. Intravenous methylprednisolone (IVMP) has been found effective in limiting morbidity and reversing the side effects caused by ipilimumab [3, 8-9]. Our patient received IVMP followed by oral tapering doses of prednisone. The patient started improving after initiating intravenous (IV) steroids and he became fully functional by the end of two months.

## Conclusions

Baseline values from laboratory analysis and a detailed physical exam should be performed and noted before beginning therapy with ipilimumab. Additionally, adequate on-treatment monitoring protocols and after-treatment follow-up protocols should be established to mitigate the risk of developing rare irAEs. Rare adverse events are very likely to go unnoticed because the degree of suspicion remains low among clinicians. Clinicians should be aware of this rare but life-threatening immune-related adverse reaction to ipilimumab, especially when the drug has been increasingly used to treat metastatic melanoma.
